# Adenosine in the Thymus

**DOI:** 10.3389/fphar.2017.00932

**Published:** 2017-12-22

**Authors:** Krisztina Köröskényi, Gergely Joós, Zsuzsa Szondy

**Affiliations:** ^1^Department of Biochemistry and Molecular Biology, Faculty of Medicine, University of Debrecen, Debrecen, Hungary; ^2^Department of Basic Medical Sciences of Dental Faculty, University of Debrecen, Debrecen, Hungary

**Keywords:** efferocytosis, adenosine, thymocyte, apoptosis, T cell selection

## Abstract

Adenosine is an ancient extracellular signaling molecule that regulates various biological functions via activating four G-protein-coupled receptors, A_1_, A_2A_, A_2B_, and A_3_ adenosine receptors. As such, several studies have highlighted a role for adenosine signaling in affecting the T cell development in the thymus. Recent studies indicate that adenosine is produced in the context of apoptotic thymocyte clearance. This review critically discusses the involvement of adenosine and its receptors in the complex interplay that exists between the developing thymocytes and the thymic macrophages which engulf the apoptotic cells. This crosstalk contributes to the effective and immunologically silent removal of apoptotic thymocytes, as well as affects the TCR-driven T-cell selection processes.

## Introduction

Generation of immunocompetent T lymphocytes from bone marrow-derived CD4^-^CD8^-^ double negative progenitors takes place in the thymus. This process occurs through a series of differentiation and selection steps (Bommhardt et al., 2004) leading via apoptosis to the removal of 95% of thymocytes produced with improper TCR. T-cell differentiation is unique in a sense that it is not a cell-autonomous process, but depends on signals provided by fibroblasts, thymic epithelial cells, macrophages, dendritic cells, and endothelial cells that surround the developing thymocytes (Petrie and Zuniga-Pflucker, 2007). Among these cells thymic epithelial cells play the major role in shaping the T cell repertoire by presenting self-antigens on their cell surface (Alexandropoulos and Danzi, 2012), while thymic F4/80^+^ macrophages are the main cleaners of the constantly produced dying cells (Surh and Sprent, 1994). While generally tissue resident macrophages are originated from the yolk sac (Gomez et al., 2015), part of the thymic macrophages seem to be differentiated from the hematopoietic stem cells (Haymaker et al., 2012) or even from T cell progenitors (Esashi et al., 2004) in the bone marrow.

At the beginning of differentiation, CD4^-^CD8^-^ progenitor cells rearrange their TCR β gene locus to produce a pre-TCR complex. Subsequently, thymocytes divide rapidly, become CD4^+^CD8^+^ DP thymocytes, then they rearrange their TCRα chain as well. Those thymocytes, which successfully generate a functional α chain replace the pre-TCR with mature TCRαβ and continue to differentiate CD4^+^CD8^+^ TCRαβ^low^ cells (Hernandez et al., 2010). The TCR of these thymocytes is able to interact with self-peptides presented on major histocompatibility complex molecules of thymic non-lymphoid cells. Their destiny will be decided by the strength of the TCRαβ signal generated during this interaction. Cells receiving a TCRαβ signal of intermediate strength continue to differentiate into CD4^+^ or CD8^+^ TCRαβ^high^ single positive thymocytes, leave the thymic cortex and complete their maturation in the thymic medulla, while those, which are exposed to a strong TCRαβ signal, are eliminated by activation-induced apoptosis, a major mechanism for generating self-tolerance (Kisielow and von Boehmer, 1995). A few percent of thymocytes that have TCR specificities for higher affinity ligands than that of the conventional CD4^+^ T cells (Moran et al., 2011) are diverted into CD4^+^CD25^+^FoxP3^+^ tT_reg_, which induce peripheral tolerance by silencing excessive peripheral immune responses, thus prevent autoimmunity (Benoist and Mathis, 2012). The remaining 90% of the DP thymocytes express TCRs, which cannot interact with peptide-loaded self-major histocompatibility molecules, thus in the absence of TCR-driven cell survival signals they undergo a default apoptosis cell death pathway named “death by neglect.”

Increasing evidence indicate that molecules produced by macrophages during the constant engulfment of apoptotic cells generate a thymic milieu for the developing thymocytes that affect the TCR-determined cell selection processes. These molecules are produced primarily not to regulate thymic selection processes, rather they are needed for the macrophages themselves. Thus, in response to the chemotactic signals produced by dying thymocytes, macrophages release ATP that contributes to their migration to the apoptotic site (Kronlage et al., 2010). Following apoptotic cell phagocytosis, they release TGF-β and prostaglandin E_2_ (PGE_2_) to prevent their pro-inflammatory cytokine production in an autocrine manner (Fadok et al., 1998). During degrading hem containing proteins, they also produce CO as a result of hem oxygenase-I action (Gemsa et al., 1973). And finally, macrophages also produce retinoids in an engulfment-dependent manner (Garabuczi et al., 2013) that regulate their own phagocytic capacity (Sarang et al., 2014). However, DP thymocytes express receptors for the macrophage-released molecules, such as ATP, PGE_2_, TGF-β, CO or retinoids (Cillie et al., 1983; Suzuki et al., 1991; Szondy et al., 1997; Levéen et al., 2005; Tsukimoto et al., 2006; Dzhagalov et al., 2007), and respond to them with an outcome that depends on the simultaneous TCR signaling. Thus, all these molecules were shown to induce or enhance thymocyte death in the absence of TCR signaling (Szondy et al., 2012). Macrophage-derived retinoids and TGF-β also synergize in the induction of TG2 (Fésüs and Szondy, 2005) in apoptotic thymocytes (Garabuczi et al., 2013). TG2 in dying cells contributes to the formation of a chemotactic signal for macrophage recruitment (Nishiura et al., 1998), prevents the leakage of the harmful cell content (Piredda et al., 1997) and facilitates the exposure of phosphatidylserine, the main important apoptotic cell recognition signal for macrophages (Sarang et al., 2007). On the other hand, in the presence of TCR signaling retinoids inhibit TCR-mediated cell death in a dose-dependent manner, thus regulate the threshold of negative selection (Szondy et al., 1998; Szegezdi et al., 2003; Sarang et al., 2013), while TGF-βplays such a determining role in the formation of tT_reg_ cells, that no tT_reg_ cells can be detected in the thymus until apoptosis and the consequent engulfment-dependent TGF-β production is induced (Liu et al., 2008; Konkel et al., 2014; Chen and Konkel, 2015).

The fact that adenosine is also produced in the thymus and affects thymic selection processes was discovered after it was found that ADA deficiency leads to adenosine accumulation and severe T cell deficiency (Apasov et al., 2000).

## Adenosine

Adenosine is an intra- and extracellularly produced purine nucleoside, which concentration is strictly controlled due to its intense and diverse biological effects. Intracellularly, it can be produced by three main pathways: (a) decomposition of adenine nucleotides (ATP, ADP, AMP) by ATPases, ATPase and cellular 5′ nucleotidase (c5′-NT); (b) hydrolysis of *S*-Adenosyl-L-homocysteine by its hydrolase; (c) phosphodiesterase-mediated degradation of cAMP (Park and Gupta, 2012). Intracellularly ADO can be converted to inosine by ADA and later on to uric acid (da Rocha Lapa et al., 2014). It also can be shifted back to the nucleotide pool in the form of AMP by ADO kinase (Ramakers et al., 2011). Finally, it can be transported to the extracellular space by specific transporters (Antonioli et al., 2014). The extracellular ADO concentration is lower than 1 μM (30–200 nM) under physiological conditions, but it can reach extremely high levels (>100 μM) during inflammation and hypoxia (Haskó and Cronstein, 2004). The waste majority of extracellular ADO is formed extracellularly from ATP, which is transported out from various cells via exocytosis, co-release or ion channels (Chen et al., 2013; Antonioli et al., 2014). Extracellular ATP then undergoes a two-step hydrolysis. The rate limiting step in extracellular ADO formation is catalyzed by either ecto-nucleoside triphosphate diphosphohydrolases, such as CD39 that phosphohydrolyses ATP, and less efficiently ADP, in a Ca^2+^- and Mg^2+^-dependent fashion, to yield AMP (Heine et al., 2001), or by members of the ecto-nucleotide pyrophosphatase/phosphodiesterase family, such as NPP1 and 3, which are also located on the cell surface, but produce AMP directly (Stefan et al., 2006). AMP in turn, is rapidly degraded to ADO by soluble or membrane-bound ecto-5′-nucleotidases, such as CD73 (da Rocha Lapa et al., 2014). The effect of extracellular ADO is terminated by the decomposition of ADO by ecto-ADA or by the uptake into the surrounding cells through equilibrative nucleoside transporters (Liu and Xia, 2015).

## The Different Adenosine Receptors

The regulatory effects of ADO, are almost exclusively mediated by the activation of its plasma membrane-associated receptors (ADO receptors) (Fredholm et al., 2001) which are expressed in a cell type specific manner. All of the four ARs (A_1_R, A_2A_R, A_2B_R, A_3_R) belong to the large family of seven-transmembrane receptors, also known as G-protein-coupled receptors (Fredholm et al., 2001). Physiological ADO levels activate A_1_R, A_2A_R, and A_3_R receptor subtypes (K_i_ = 100–300 nM for human receptors). A_2B_Rs (K_i_ = 15 μM for human A_2B_R) are activated only during pathological conditions (e.g., hypoxia, inflammation) (Haskó et al., 2007; Eltzschig, 2009; Dubyak, 2012). The duration and magnitude of the ADO’s effect are influenced by a number of processes including intra- and extracellular production of ADO, transport, cellular uptake, degradation, receptor density, as well as the activity of receptors and signaling molecules (Eltzschig, 2009; Chen et al., 2013). In general, ARs affecting intracellular cAMP levels are involved in the regulation of a large scale of biological processes (Liu and Xia, 2015). In addition, all of the four ARs can activate different types of MAPKs (p38, ERK1/2, JNK) (Antonioli et al., 2014). Further AR downstream effectors include phospholipase C, ion channels, phosphoinositide 3-kinase and protein kinase B, nitric oxide, reactive oxygen species, and lipid mediators (Liu and Xia, 2015). Like other G-protein-coupled receptors, ARs tend to form homo- and heterodimers as well as oligomers (Chen et al., 2013). With the formation of such complexes, the range of signaling pathways and biological processes controlled by ARs is further broadened.

## Adenosine Metabolism and Adenosine Receptors in the Thymus

Studies in our laboratory have found that neither macrophages nor apoptotic thymocytes produce detectable amount of ADO, if they are cultured alone, but ADO is produced during the apoptotic cell engulfment indicating that ADO is an additional molecule which appears in the thymic milieu in an engulfment-dependent manner (Köröskényi et al., 2011). Apoptotic cells release adenine nucleotides via pannexin channels (Chekeni et al., 2010; Yamaguchi et al., 2014), which open in a caspase-dependent manner (Sandilos et al., 2012). In addition, thymocytes release ATP also via a pannexin channel-independent manner, when they undergo secondary necrosis (Sándor et al., 2017). CD39 is constitutively expressed in the thymus and is associated primarily with macrophages and tT_reg_ cells (Antonioli et al., 2013), while thymocytes express NPP1 (Petersen et al., 2007). Thus in the thymic cortex, where apoptotic thymocytes and macrophages are present in close proximity, both cell types can contribute to the extracellular degradation of ATP to AMP. In accordance, high amount of AMP was detected in the culture of dying thymocytes (Yamaguchi et al., 2014). However, while CD73 and prostatic acid phosphatase are expressed by macrophages (Eichin et al., 2015), in thymocytes CD73 expression is differentiation-dependent: it is virtually absent in cortical thymocytes, but becomes upregulated in the medullary ones (Resta et al., 1997, 1998). Thus in the thymic cortex, where the majority of improper thymocytes die, ADO formation must be fully dependent on the CD73 activity of macrophages. Indeed, in the context of dying thymocytes and engulfing macrophages ADO production was confirmed *in vitro* to be fully dependent on the CD73 activity of macrophages (Yamaguchi et al., 2014; Sándor et al., 2017). The endogenous CD73 activity of macrophages is so high in the thymus that even if CD73 expression is artificially upregulated in thymocytes, the *in vivo* thymic ADO levels do not change (Resta et al., 1997). The extracellular ADO concentrations in the thymus, however, are determined by the thymocyte ADA activities as well, and ADA activity was found to be extraordinarily high in cortical thymocytes, while to be strongly downregulated in the medullary ones (Chechik et al., 1981; Ma et al., 1982). These observations indicate that either ADO concentrations are kept lower in the cortical zone, where most of the positive selection takes place, while are higher in the medullary zone, where selected thymocytes mature further and also undergo negative selection. Alternatively, since in the medullary zone the rate of apoptosis, consequently the rate of ATP release is lower, thymocyte cell surface CD73 expression and the low thymocyte surface ADA activity together maintain the sufficient ADO levels around the thymocyte ARs. Loss of ADA activity in ADA deficient patients is associated with increased ADO levels in the thymus, which thus affect primarily the cortical thymocytes (Resta and Thompson, 1997). ADA, however, is responsible for the degradation of deoxyadenosine as well, thus low ADA activities in the cortex might promote formation of the intracellular dATP and consequently proliferation of cortical thymocytes (Sandberg, 1983). ARs are expressed already by the T-lymphopoietic and monocytopoietic cells as well, and both of them express all the four ARs. While, however, in the T-lymphopoietic lineage the expression of A_2A_Rs dominates, in the monocytopoietic cells all the A_2A_, A_2B_, and A_3_ receptors are highly expressed subtypes (Streitová et al., 2010). However, as thymic apoptosis is initiated in the developing thymus and phagocytosis follows, engulfing macrophages upregulate their A_2A_Rs (Köröskényi et al., 2011), while downregulate the A_3_ ones (Duró et al., 2014). Upregulation of the A_2A_Rs is engulfment-dependent in macrophages, and involves both lipid sensing transcription factors and CO produced via the hem oxygenase reaction (Haschemi et al., 2007; Köröskényi et al., 2011).

## Effect of Adenosine on the T Cell Development

Early studies on fetal thymic organ cultures conducted in the presence of the ADO agonist 5′-(*N*-ethyl)-carboxamidoadenosine, and the AR antagonist 8-phenyl-theophylline (8-PT) indicated that ARs influence the development of thymocytes, since administration of both compounds resulted in a decreased thymocyte cell number (Hamad, 1997). Results obtained with 8-PT were concluded in a way that ADO is required for proper thymocyte development, while results obtained with NECA indicated that higher concentrations of it induce thymocyte death. 8-PT is, however, not only an AR antagonist, but acts also as a potent inhibitor of cAMP phosphodiesterase. Thus, it can induce elevations in the intracellular cAMP levels (Nicholson and Wilke, 1989), and a consequent loss of thymocyte cell number due to cAMP-induced thymocyte death (McConkey et al., 1990). Thus 8-PT could decrease the thymocyte cell number by inducing apoptosis, rather than ADO being required generally for thymocyte differentiation. In accordance, loss of A_2A_Rs, the dominant thymocyte AR subtype, does not affect the number and the distribution of various thymocyte populations in mice (Kiss et al., 2006), despite of the fact that the expression of other ARs remained unchanged (Lukashev et al., 2003). ADO is, however, constantly produced in the cortical layer of the thymus, and can contribute to the apoptosis of neglected thymocytes similar to other signaling molecules that are also produced by macrophages in an engulfment-dependent manner (Szondy et al., 2012). The ADO-induced death of mouse thymocytes is strongly dependent on the A_2A_Rs, as in A_2A_R null thymocytes ADO fails to induce significant cell death (Kiss et al., 2006). The ADO-dependent death in mouse thymocytes is mediated via cAMP and cAMP-dependent protein kinase (Armstrong et al., 2001; Kiss et al., 2006) and involves upregulation of Bim, a BH3-only pro-apoptotic member of the Bcl-2 protein family (Kiss et al., 2006). In human thymocytes, on the other hand, ADO induces a Ca^2+^-dependent death (Szondy, 1994). ADO also promotes the glucocorticoid-induced death of neglected thymocytes (McConkey et al., 1993), and together with the engulfing macrophage-produced retinoids and TGF-β, also contributes to the upregulation of TG2 in the dying cells (Sándor et al., 2016, 2017). Thus, engulfing macrophage-produced apoptosis-promoting molecules, including ADO, appearing in the cortical thymic milieu cooperate in both the fast killing of thymocytes, which express useless TCRs, and in the upregulation of their TG2 expression. These signals together are so crucial for the upregulation of TG2 *in vivo* that *in vitro,* though thymocytes die upon exposure to apoptotic stimuli, they cannot upregulate the expression of the protein (Szegezdi et al., 2000). Several molecules, which induce apoptosis of neglected thymocytes, also interfere with the TCR-mediated signaling pathways and inhibit negative selection in a dose dependent manner (Ashwell et al., 1996; Szondy et al., 1998; Szegezdi et al., 2003). For glucocorticoids, it has been demonstrated that this way these molecules determine the TCR avidity thresholds that determine whether developing thymocytes survive or die, and therefore help to mold the antigen-specific T cell repertoire (Ashwell et al., 1996). Interestingly, ADO has also been reported to interfere with the TCR-induced signaling pathways and negative selection via interfering with the CD3 ζ chain phosphorylation (Apasov et al., 2000). Thus ADO, similar to glucocorticoids and retinoids, might affect the TCR avidity thresholds in a dose dependent manner. And finally, development of tT_reg_ cells have been shown to be dependent on engulfing macrophage-derived TGF-β (Konkel et al., 2014; Chen and Konkel, 2015). Interestingly, both retinoids (Mucida et al., 2007) and ADO (Ohta and Sitkovsky, 2014) promote the TGF-β-dependent development of T_reg_ cells in the periphery. In CD73/prostatic acid phosphatase double knock out mice, where the extracellular ADO levels are low, the tT_reg_ cell production was found to be impaired (Yegutkin et al., 2014) indicating the involvement of ADO also in the tT_reg_ cell differentiation.

## Effect of Adenosine on the Engulfment-Related Processes of the Macrophage

Apoptotic cells release find me signals for macrophages so that the macrophages could find them efficiently. In the thymic cortex the apoptosis sensitive DP thymocytes are in strong interaction with the cortical macrophages forming clusters with them (Rezzani et al., 2008). Thus, the removal of the constantly appearing apoptotic cells might not require chemotactic migration of macrophages. When, however, a large amount of DP thymocytes die, additional macrophages migrate through vessels into the thymus, and enter the cortical region, where most of the apoptosis takes place (Odaka and Mizouchi, 2002). In macrophages a purinergic autocrine signaling functions to amplify and translate chemotactic signals into directional motility (Kronlage et al., 2010). Upon sensing chemotactic signals, macrophages release ATP at the leading edge of the cell, which is then degraded to ADP, AMP, and ADO. Loss of either adenosine A_3_R or A_2B_R receptor function blocks the chemotactic migration of macrophages toward the apoptotic cells (Joós et al., 2017). The involvement of AR subtype in the control of chemotactic navigation, however, might be macrophage type specific, since peritoneal tissue resident macrophages express both receptors at high levels, while in monocyte-derived macrophages the expression of the adenosine A_2B_R dominates. In accordance, loss of A_3_Rs affects the apoptotic cell removal in the peritoneal cavity, but has no effect on the thymic apoptotic cell removal *in vivo* (Joós et al., 2017). ADO has no effect on the phagocytosis of apoptotic cells (Köröskényi et al., 2011; Duró et al., 2014), but it might contribute to the apoptotic cell-induced upregulation of cell surface TG2 (Szondy et al., 2003; Rébé et al., 2009; Sarang et al., 2014) which acts as a phagocytosis coreceptor in macrophages (Tóth et al., 2009) and contributes to the activation of TGF-β released in latent form (Nunes et al., 1997). ADO is also required to maintain the immunologically silent removal of apoptotic cells. In the absence of A_2A_R signaling KC and macrophage inflammatory protein-2 neutrophil chemokines are released by engulfing macrophages (Köröskényi et al., 2011). During the engulfment of apoptotic cells several anti-inflammatory mechanisms are activated to prevent the production of pro-inflammatory cytokines (Trahtemberg and Mevorach, 2017). Many of them act via inhibiting nuclear factor kappa-light-chain-enhancer of activated B cells (NF-κB) transcriptional activity which plays a key role in the induction of inflammatory cytokine genes (Baeuerle and Henkel, 1994), or via inducing the production of TGF-β or IL-10 (Szondy et al., 2017). ADO signaling was also shown to interfere with the NF-κB function by inhibiting its nuclear translocation, DNA binding and transcriptional activity (Xia et al., 2000; Majumdar and Aggarwal, 2003; Lukashev et al., 2004) and to induce IL-10 (Németh et al., 2005). In addition, A_2A_R signaling prevents nitrogen monoxide formation in engulfing macrophages (Köröskényi et al., 2011) and enhances the expression of dual specific phosphatase, which interferes the MAPK signaling pathways known to contribute also to the pro-inflammatory cytokine expression (Köröskényi et al., 2016). A_3_Rs, on the other hand, were found to promote apoptotic cell uptake-induced neutrophil chemoattractant formation (Duró et al., 2014). Since A_3_Rs are downregulated during engulfment, while the expression of A_2A_Rs is induced, the immune silencing signaling of ADO acting via the upregulated A_2A_Rs dominate during phagocytosis of apoptotic cells.

## Conclusion

**FIGURE 1:**
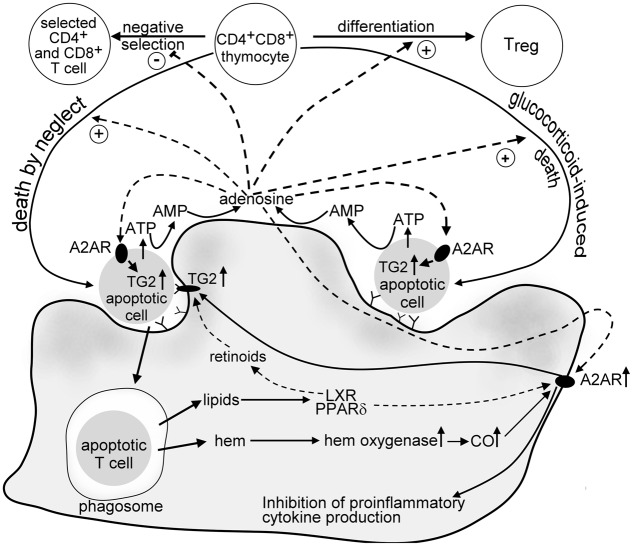
Suggested crosstalk between developing thymocytes and engulfing macrophages in the thymus involving adenosine. Dying thymocytes release ATP via caspase-regulated pannexin channels. ATP is then fast degraded to AMP by cell surface ATP degrading enzymes of thymocytes and macrophages, and to adenosine by CD73 expressed on the macrophage cell surface. ADO acting on thymocyte adenosine A_2A_ receptors induces “death by neglect” alone or promotes the glucocorticoid-induced death of DP thymocytes. In addition, it interferes with the negative selection of thymocytes that have TCR specificities with intermediate affinity for self-antigens, thus promote positive selection. ADO is also required for the tTreg formation. In dying thymocytes ADO enhances the intracellular expression of TG2, an apoptosis-related protein that promotes fast recognition of apoptotic cells by macrophages. In macrophages ADO activates adenosine A_2A_ receptors, the expression of which is strongly induced following apoptotic cell uptake. The mechanism involves both hem oxygenase and the lipid sensing peroxisome proliferator activating receptor δand liver X receptor that are triggered by the fatty acid and oxysterol content of the engulfed apoptotic cells, respectively. A_2A_ adenosine receptor signaling in macrophages prevents neutrophil chemokine formation and might also contribute to the apoptotic cell lipid content-induced upregulation of cell surface TG2. TG2 in macrophages acts as a phagocyte coreceptor for the proper phagocytosis of apoptotic cells, and contributes to the activation of latent TGF-β, an anti-inflammatory cytokine released by the engulfing macrophages.

Increasing evidence indicate that macrophages engulfing apoptotic cells respond to the chemotactic signals released by apoptotic cells, to the apoptotic cell engagement and to the apoptotic cell uptake with producing various molecules, such as ATP, IL-10, TGF-β, CO, prostaglandin E_2_, retinoids, and also ADO. These molecules together regulate either the phagocytic activity of macrophages and/or contribute to the immunologically silent removal of apoptotic cells. However, they might play also additional roles in the maintenance of tissue homeostasis, and this role vary from tissue to tissue. The data presented in this review indicate that in the thymus ADO in an interplay with other engulfing macrophage-derived molecules might contribute to the thymocyte selection processes (**Figure 1**).

## Author Contributions

All authors listed have made a substantial, direct and intellectual contribution to the work, and approved it for publication.

## Conflict of Interest Statement

The authors declare that the research was conducted in the absence of any commercial or financial relationships that could be construed as a potential conflict of interest.

## Abbreviations

ADA: adenosine deaminase
ADO: adenosine
AR: adenosine receptor
CO: carbon monoxide
DP: double positive
MAPK: mitogen-activated protein kinase
NPP: nucleotide pyrophosphatase/phosphodiesterase
NECA: *N*-ethyl-carboxamidoadenosine
8-PT: 8-phenyl-theophylline
PGE_2_: prostaglandin E_2_
TCR: T-cell receptor
TG2: transglutaminase 2
TGF-β: transforming growth factor-β
T_reg_: regulatory T cell
tT_reg_: thymic regulatory T cell
